# Wettability-Oriented Laser Microgrooving Process on Cemented Carbide Surface

**DOI:** 10.3390/ma17143423

**Published:** 2024-07-11

**Authors:** Jing Ni, Xianle Huang, Zhen Zhang, Zuji Li, Binjie Lv, Xinyu Gao

**Affiliations:** School of Mechanical Engineering, Hangzhou Dianzi University, Hangzhou 310018, China; nj2000@hdu.edu.cn (J.N.); 202241010001@hdu.edu.cn (X.H.); 222010116@hdu.edu.cn (Z.L.); 21110427@hdu.edu.cn (B.L.); 222010055@hdu.edu.cn (X.G.)

**Keywords:** laser scanning, groove texture, wettability, cemented carbide

## Abstract

Surface micro-texture has been shown to enhance wettability and reduce wear on cutting tools. However, there is limited research on how laser parameters impact the dimensional accuracy of surface texture and its wettability. This study focuses on producing arrayed groove textures on WC/Co cemented carbide surfaces using Nd: YAG laser, evaluating the effect of the laser parameters on surface topography and texture accuracy through microscopic observation and simulation. The results indicate that, with laser parameters such as a number of passes less than 5, approximately 16 W power, scanning speed of 100–150 mm/s, and pulse frequency of 30 kHz, the error between the groove width and laser spot diameter was 4.7%. Additionally, the study explores the impact of the groove texture on surface wettability using the solid droplet method and XPS analysis. Comparative experiments reveal that increased surface roughness enhanced oleophobicity, with surfaces exhibiting high texture accuracy and integrity showing improved oleophobic and spreading properties. Thus, the precise regulation of laser processes is crucial for maintaining surface texture integrity and enhancing surface wettability.

## 1. Introduction

In recent years, with the popularization and application of difficult-to-machine materials such as titanium alloys and superalloys, new challenges and requirements have been put forward for cutting processing, and various new tool structures and materials have emerged. From the perspective of the economic benefits of titanium alloy machining, cemented carbide is still the main cutting tool material in titanium alloy machining. Therefore, it is of great significance to improve the lubrication state between the tool and the workpiece, enhance the lubrication performance of the tool surface, reduce the friction, so as to reduce the cutting force and cutting heat, and prolong the service life of cemented carbide tools. Studies [1,2,3,4] have shown that micro-texture can change the wettability of the material surface and enhance the penetration of cutting fluid into the tool surface, thus improving the lubrication performance of the tool surface.

Surface micro-texture processing techniques are typically classified as conventional, unconventional, and hybrid processing methods [5]. Conventional processes include micro-milling, micro-turning, and micro-drilling using micro-tools [6]. Unconventional methods involve achieving microstructural machining without direct contact between the tool and workpiece, utilizing energy loading from sources like ultrasound, laser, and sparks [7,8]. Hybrid methods combine two or more conventional or unconventional techniques for surface micro-texture processing [9]. Laser micromachining, as an unconventional method, offers advantages such as non-contact operation, high precision, and efficiency [10]. Various types of lasers, including CO_2_, Nd: YAG, and fiber lasers, are used for laser processing, with Nd: YAG lasers being widely preferred for their high peak power [11].

Currently, a large number of studies have shown that surface texture can indeed change the wetting and diffusion behavior of droplets on the substrate [12]. However, so far, the research on the hydrophobicity of surface texture mainly focuses on the influence of the surface texture morphology on wettability and its mechanism. For example, Tong et al. [13] found that the hydrophobicity of the sample surface could be regulated by changing the dimensions of the dimple weave. Minghua Pang et al. [14] proved that the texture shape and area ratio have a coupling effect on the surface contact angle. Li Haoyue [15] has also proven that using laser processing to change the depth of the grid texture on the TC4 substrate has a great influence on the improvement of wettability. Basil Kuriachen [16] verified that the change in surface roughness induced by EDM microstructures affects the surface wettability. Similarly, Yang et al. [17] processed three different micro-textures on the surface of Inconel 718 alloy, and found that the change in surface hydrophobic behavior was caused by the combined action of surface morphology and surface chemistry.

Few researchers have studied the effect of micro-texture accuracy on surface wettability. Tomáš Primus et al. [18] changed the surface wettability of Ti6Al4V alloy by using fast nanosecond laser micro-structure process, and proposed that surface elements are more important than the micro-texture morphology. A. Riveiro et al. [19] have determined the effect of CO_2_ laser-processing parameters on surface wetting characteristics for processing PTFE surface textures and found that tiny filaments on the surface of the weave can contribute to the enhancement of hydrophobicity. F. Peter Prakash et al. [20] have changed the processing parameters of the Nd: YAG laser, affecting the overlapping area of the laser and, thus, affecting the roughness of the texture surface, and finally improving the hydrophilicity of the C-263 based nickel superalloy surface. Jian Ma et al. [21] proposed three laser-scanning strategies to achieve sinusoidal textured surfaces and discussed the effects of surface structure and chemical composition evolution on surface wettability. Under the action of hot capillary migration, the laser-induced wetting mechanism is different from the isothermal wetting mechanism.

However, there is a gap in the research regarding how the surface micro-texture of WC/Co cemented carbide affects the wetting and diffusion behavior of reactive wetting systems under laser heating conditions. This study focused on creating groove textures on the surface of WC/Co cemented carbide and assessing the impact of laser parameters such as scan time, laser power, scan speed, and pulse frequency on surface morphology and texture accuracy through microscopic observation and simulation. Additionally, the study investigated the influence of groove texturing on the surface wettability of WC/Co cemented carbide using the solid droplet method.

## 2. Experiments Details

### 2.1. Materials

In this paper, WC/Co cemented carbide (WC + 8% Co) with a size of 16 × 10 × 5 mm is used as the matrix material. The mechanical properties are shown in Table 1. Before the texturing treatment, the substrate surface was first polished, and then ultrasonic cleaning and drying were performed to remove the surface fouling. Before laser ablating, the initial surface roughness of the WC/Co cemented carbide sample surface was Sa = 17 nm.

### 2.2. Preparation of Texture

**Figure 1:**
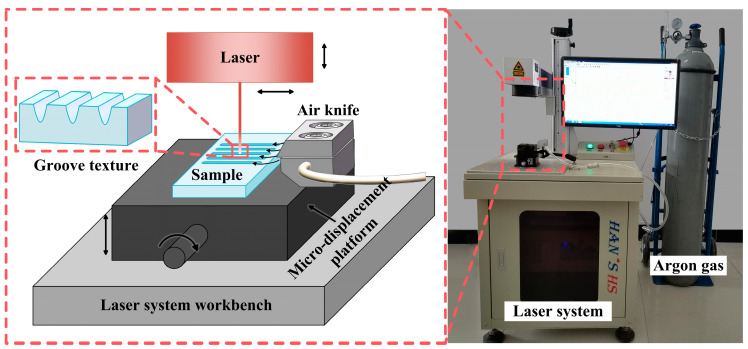
Laser-scanning device diagram.

Groove textures were designed on the surface of the polished sample, and a groove-shaped texture was prepared using a Han’s H20 fiber laser marking machine (YLP-H20, laser spot diameter 50 μm, Han’s Laser Technology Industry Group Co., Ltd., Shenzhen, China) under different laser-scanning parameters (Figure 1). In addition, a high-pressure inert gas (99.9998% argon) is used to remove wear debris from the manufactured textured surface in a dry state, and to suppress adverse chemical reactions on the surface during scanning.

### 2.3. Description of Working Conditions

#### 2.3.1. Experimental Group Planning and Morphology Measurement

Laser scanning uses a single-factor experiment to change all four factors: *N* (number of passes), *v* (speed), *P* (laser power), and *f_p_* (pulse frequencies). In order to facilitate the collection of microgroove structure information (depth and width), a set of basic processing parameters must be set to ensure the integrity of each microgroove in the experiment. The processing parameters are shown in Table 2.

The morphology of textured surface and traditional polished surface was observed by using a super-depth-of-field camera (VH-100) and a three-dimensional confocal interference profilometer (S neox, Sensofar, Terrassa, Spain). The width and depth of 20 groups of different groove textures were measured, and then the average value was calculated.

#### 2.3.2. Measurement of the Static Contact Angle

The contact between oil droplets and laser-treated surface samples was assessed using the sessile drop technique [22]. When a liquid drop is placed on a solid surface, it will assume a spherical shape that is truncated at the solid interface, forming a contact angle (CA) between the solid surface and the tangent of the sphere. Typically, the contact angle is employed to determine the hydrophilicity (CA < 90°) of a surface, with superhydrophilic/superhydrophobic surfaces exhibiting contact angles of less than 10° or greater than 150°, respectively.

When determining the contact angle of a uniform droplet volume placed on a stationary surface that is not tilted, it is commonly referred to as a static contact angle (SCA). During the measurement, 10 μL of castor oil droplets were delivered to the sample using a Carent^®^S microsyringe (adjustable volume 10 μL–1 mL, Hamilton Laboratory Equipment Co., Ltd., Shanghai, China). In the rest of this article, if the volume of the droplet is not indicated, it is 10 μL. The droplet was photographed using a contact angle measuring instrument (JC2000D1, Shanghai Zhongchen Digital Technology Equipment Co., Ltd., Shanghai, China) to observe the static contact angle of the oil droplet on the texture surface and its spreading changes. The droplet image has been analyzed by software and the SCA value was calculated. The SCA values given in this work were obtained by averaging several contact angles of different parts of the textured region.

### 2.4. Machines and Devices

#### 2.4.1. X-ray Photoelectron Spectroscopy

Equipped with elemental analysis using X-ray photoelectron spectroscopy (XPS) instruments, Thermo Scientific K-Alpha XPS (Thermo Fisher Scientific, Waltham, MA, USA) was used to evaluate the chemical composition changes of laser surface texture changes. Using a monochromatic aluminum Kα ray source, an appropriate amount of sample was pressed/cut and attached to the sample plate. The specimen was positioned in the sample chamber of the Thermo Scientific K-Alpha XPS instrument. Once the pressure in the sample chamber dropped below 2.0 × 10^−7^ mbar, the specimen was transferred to the analysis chamber. The focal point was set at 400 μm, with an operating voltage of 12 kV and a filament current of 6 mA. The pass energy for full spectrum scanning was 150 eV, with a step size of 1 eV. For the specific test, the pass energy for narrow-spectrum scanning was 50 eV, with a step size of 0.1 eV. Finally, the Kα-ray spectrum was obtained, and the Avantage analysis software (V5.9921) was used to fit the Gaussian–Lorentz fitting of the XPS fine spectrum, and the peak position was assigned with reference to the XPS database data.

#### 2.4.2. Scanning Electron Microscopy (SEM)

Scanning electron m(SEM) is a powerful technique used to examine the surfaces of micro-organisms and other specimens. Unlike light microscopes, which have limitations due to the wavelength of light, SEM (SEM, JEOL JSM-IT700HR, Tokyo, Japan) uses a beam of electrons to achieve higher resolution. SEM works by applying kinetic energy to produce signals from interactions with electrons. It uses secondary electrons, backscattered electrons, and diffracted backscattered electrons to view crystallized elements and photons. These interactions create detailed images of the specimen’s surface.

## 3. Results and Discussion

### 3.1. Effect of Laser Parameters on Groove Texture Morphology

In order to measure the depth and width of the groove on the surface of the workpiece, a three-dimensional confocal interference profilometer was used to photograph the surface of the sample, and the width and depth of each groove on the surface of the sample were measured and recorded. The texture morphology was analyzed and the cross-sectional morphology was plotted by Sensofar analysis software (v 1.9.2).

**Figure 2:**
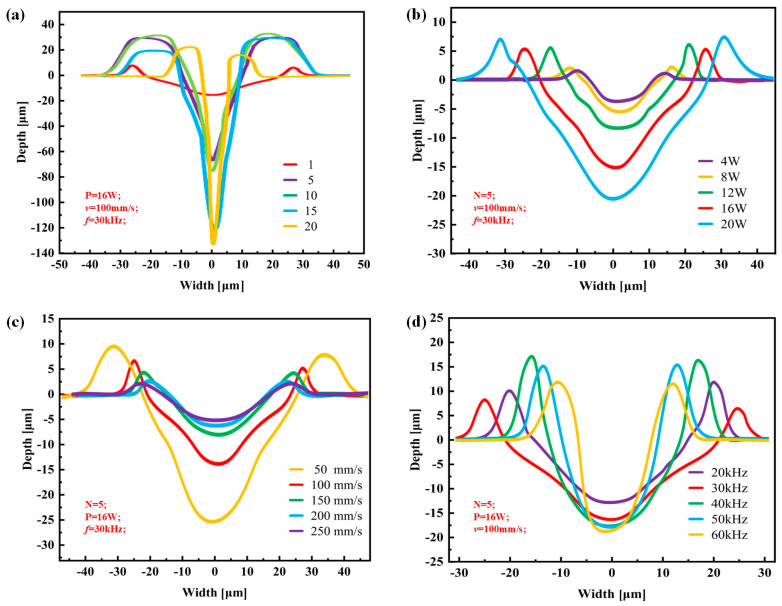
Cross-section of groove texture under different laser-processing parameters: (**a**) change in number of passes (*N*); (**b**) change in laser power (*P*); (**c**) change in scanning speed (*v*); and (**d**) change in pulse frequency (*f_l_*).

**Figure 3:**
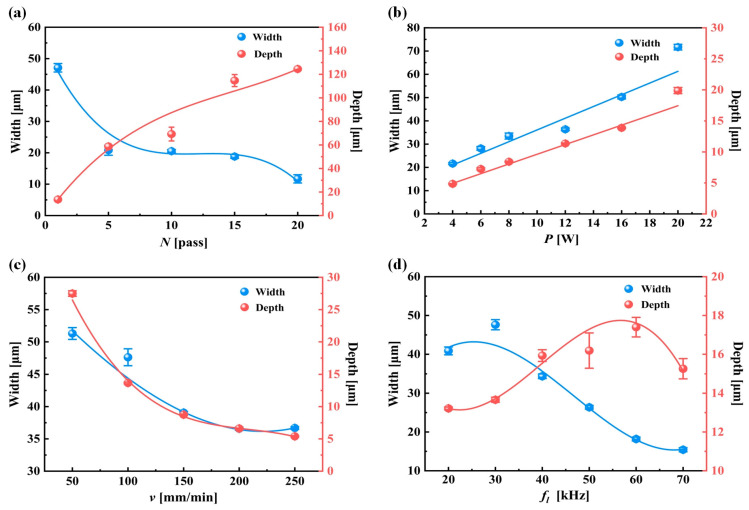
The width and depth of groove texture under different working conditions: (**a**) change in number of passes (*N*); (**b**) change in laser power (*P*); (**c**) change in scanning speed (*v*); and (**d**) change in pulse frequency (*f_l_*).

**Figure 4:**
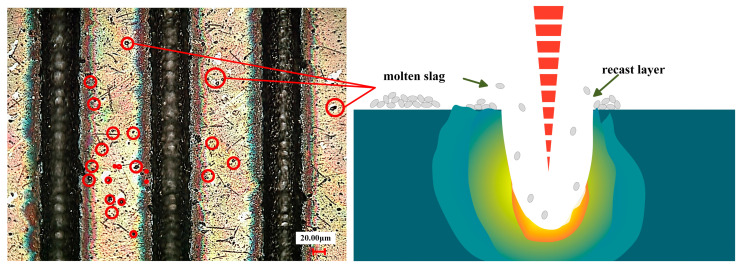
Laser-processing schematic diagram.

The width and depth of the groove texture under each set of working conditions are obtained by averaging the width and depth of different grooves under the same set of working conditions. Figure 2 shows the cross-section of the groove texture under different laser-processing parameters. The texture morphology with the same processing parameters in Figure 2 is shown by red lines. Figure 3 shows the variation in the groove texture width and depth under different working conditions. The meaning of the error bars in the graphs in Figure 3 is the variance of the data obtained for three pairs of weave widths and depths with respect to the means. Figure 4 shows a schematic diagram of the laser processing.

**Figure 5:**
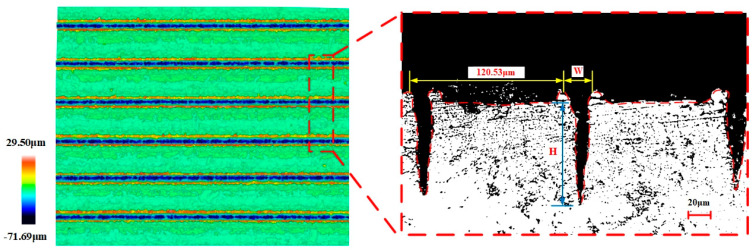
The top view of WC/Co cemented carbide texture surface and the cross-section of groove texture under 10 passes.

It can be seen from Figure 2a and Figure 3a that the power change will significantly increase the groove depth and width at the same time which is a different influence than in the case of changing the number of passes. As shown in Figure 4, this was because, when the number of processing increases, the molten pool phenomenon in the processing process causes the slag at the bottom of the groove texture to accumulate on both sides of the groove, resulting in an increase in the depth and a decrease in the width. After the number of passes reaches five, the width of the groove tends to change gently. This was because, when the depth of the groove becomes greater, the actual processing plane is far away from the laser focal plane, resulting in a gradual weakening of the laser’s ability to remove the material (the processing depth that the laser can achieve without refocusing was limited [23]), and the width at the bottom of the groove was very narrow, as shown in Figure 3a and Figure 5, resulting in a decrease in the slag in the molten pool phenomenon, making the width change not significant. On the other hand, when the processing exceeded five passes, the surface of the material will produce a more serious heat-affected zone. The temperature near the laser spot surpasses the removal temperature of the WC/Co cemented carbide material after 10 or more passes, leading to the laser-induced removal of material at the edge of the groove texture.

It can be seen from Figure 2b and Figure 3b that the power change will significantly increase the groove depth and width at the same time which is a different influence than in the case of changing the number of passes. When the laser power is too small, below 4 W, the groove morphology is discontinuous and the depth is shallow, while, when the energy exceeds 4 W, the groove morphology is generally better. When the power is 20 W, the maximum groove width is 70 μm. When the laser power reaches about 16 W, the groove width is basically the same as the laser spot size. When the power is greater than 16 W, the groove width changes faster. This is because the laser energy density determines the energy absorbed by the machined surface per unit area. The calculation formula [24] was as follows:

(1) $$E_{d}=\frac{4P}{\pi f_{l}d^{2}}$$

*E_d_*—energy density, *P*—laser power, *f_l_*—pulse frequency, and *d*—laser beam diameter.

**Figure 6:**
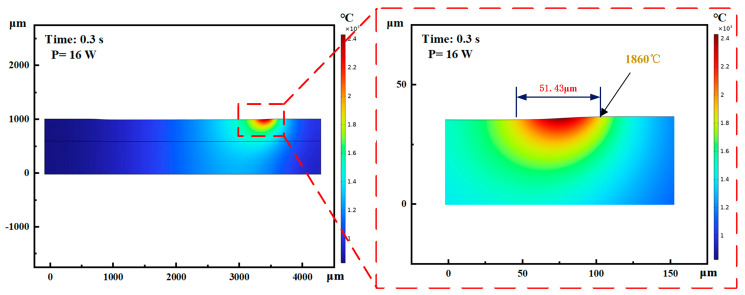
Cross-sectional temperature simulation of laser processing groove texture on WC/Co cemented carbide surface.

Because the energy density of the spot is a Gaussian distribution, the energy density of the edge part of the spot is less than the material damage threshold (1280 °C) when the power is small, and the surface of the material cannot be melted, so the actual width of the groove is less than the size of the laser spot. When the power increases, due to heat transfer, the energy density of the edge part of the spot is greater than the material surface damage threshold, and the actual width of the groove gradually increases. Therefore, the power selection should be at least greater than 8 W to ensure the quality of the groove morphology. Further, in order to ensure the accuracy of the groove width and the integrity of the groove, the laser power should be 16 W (show as Figure 6). The simulation was conducted using COMSOL software (v 6.2.0.415) with a laser light source set to a Gaussian distribution. The material parameters of WC/Co alloy, laser-processing parameters, and the moving trajectory were inputted to obtain the simulation results. The use of a larger power will lead to an increase in the heat-affected zone, with which it is easy to damage the material.

**Figure 7:**
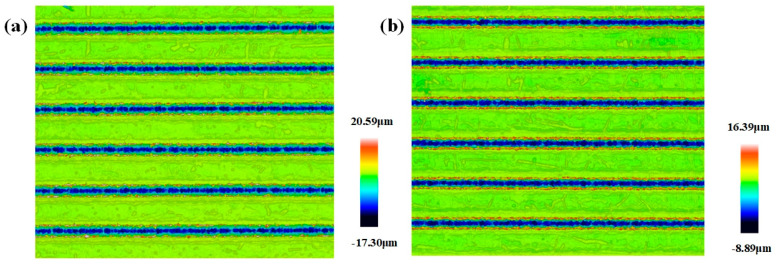
Textured surface topography at different scanning speeds: (**a**) 100 mm/min; and (**b**) 200 mm/min.

It can be seen from Figure 2c and Figure 3c that the width and depth of the microgroove texture decrease with the increase in the scanning speed. The change in the depth–width ratio is not obvious. It can be clearly found in Figure 2c and Figure 7a that the separation and coincidence of laser pulses, and the groove morphology are better when the speed is set to 100 mm/s. In Figure 7b, when the speed is greater than 200 mm/s, it is difficult to extract the effective depth information of the microgroove. Excessive speed means that the pulse energy cannot overlap, and the groove cannot be formed. This phenomenon can be explained by the following statement: In laser processing, the pulsed laser irradiates the workpiece vertically, and the three-co-ordinate workbench drives the workpiece to make a relative motion along the horizontal direction at a certain speed, thereby ablating into a groove. The speed of this relative motion is usually denoted as the laser-scanning speed. Because the distance between the pulse spots is relatively close, the coincidence degree between the spots is larger, and the energy injected at the same position is also greater. At this time, it is more suitable to use the effective pulse number to calculate this.

**Figure 8:**
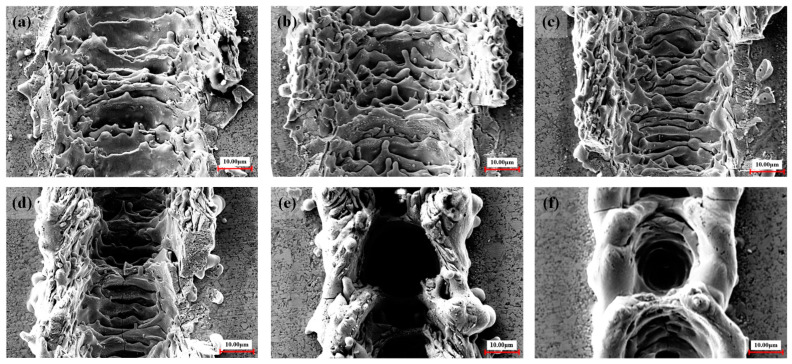
SEM photographs of groove weave morphology at different pulse frequencies: (**a**) pulse frequency of 20 kHz; (**b**) pulse frequency of 30 kHz; (**c**) pulse frequency of 40 kHz; (**d**) pulse frequency of 50 kHz; (**e**) pulse frequency of 60 kHz; and (**f**) pulse frequency of 70 kHz.

**Figure 9:**
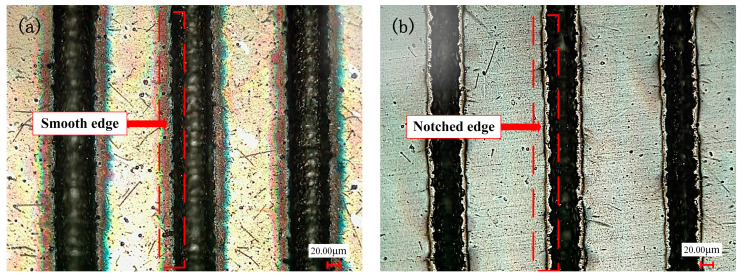
The textured surface morphology was photographed by optical microscope at different pulse frequencies: (**a**) pulse frequency of 30 kHz; and (**b**) pulse frequency of 60 kHz.

It can be seen from Figure 2d and Figure 3d that, as the frequency increases, the groove width becomes smaller (the range of variation is 47–30 μm), and the depth becomes larger (the range of variation is 11–22 μm). When the laser frequency is raised from 20 kHz to 30 kHz, the higher energy per unit time leads to a temperature increase near the edge of the laser spot, reaching the material removal threshold. This results in an expansion of the groove width. With a further increase in laser frequency, the material melts and becomes more fluid, accumulating at the groove edge and causing a gradual reduction in groove width. When the laser frequency is increased to 70 kHz, regular round pits appear in the bottom structure of the groove weave, as illustrated in Figure 8. These pits are encased in a barrier of melted carbide created by the laser. The presence of this melted carbide surrounding the round pits results in a reduction in the average measured depth of the groove structure. When the repetition frequency reaches 30 kHz, the width of the groove reaches the maximum. When the repetition frequency is too high, the plasma shielding effect will inevitably occur, making the groove edge morphology uneven, as shown in Figure 9b at a frequency of 60 kHz. In order to avoid the generation of a plasma shielding effect as much as possible, the repetition frequency should be selected as small as possible in the selection of laser-processing parameters. When the frequency is reduced, the single pulse energy was increased, which is beneficial to the microgroove forming.

Usually, when the laser beam irradiates the surface, the energy of the laser is absorbed by the surface, and it increases the temperature of the irradiated area. The increase in temperature leads to the heating, melting, evaporation, and plume formation in the impact zone. These phenomena are combined to form a cavity in the impact zone. During laser scanning, the grooves are filled with molten metal, leading to the formation of plumes. Over time, the formed plume erupts in the cavity, as shown in Figure 4, and the molten metal splashes out from the pit. Subsequently, the molten metal solidifies, forming a re-solidified layer near the texture [25].

In summary, when the number of laser processing rounds is less than five passes, the laser power is about 16 W, the scanning speed is between 100–150 mm/s, and the repetition frequency is 30 kHz, the minimum error between the width of the groove texture on the WC/Co cemented carbide surface and the diameter of the laser spot is only 4.7%, the accuracy is the highest, and the overall morphology of the groove texture is the most consistent.

### 3.2. Effect of Microstructure Size (Precision) on Wettability

**Figure 10:**
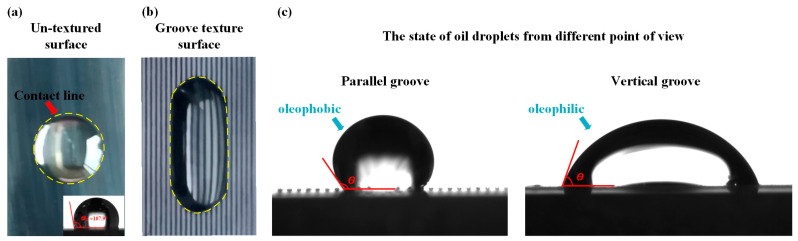
The oil drops on the surface: (**a**) top view of smooth-surface oil droplets; (**b**) top view of groove-texture-surface oil droplets; and (**c**) side view of oil droplets on groove texture surface in two directions.

**Figure 11:**
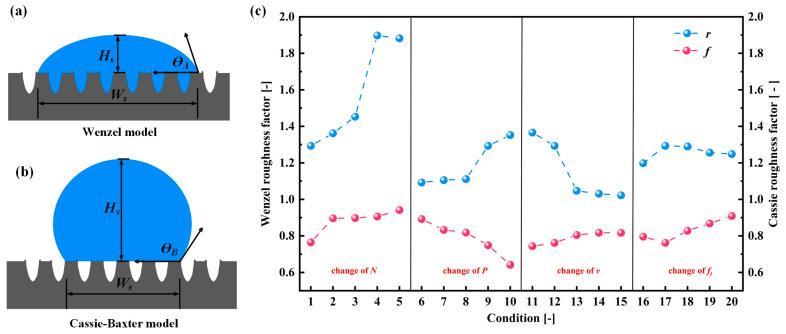
Surface roughness factor measurements: (**a**) Wenzel model schematic diagram; (**b**) Cassie–Baxter model schematic diagram; and (**c**) roughness factor from the Wenzel model and the Cassie–Baxter model.

**Figure 12:**
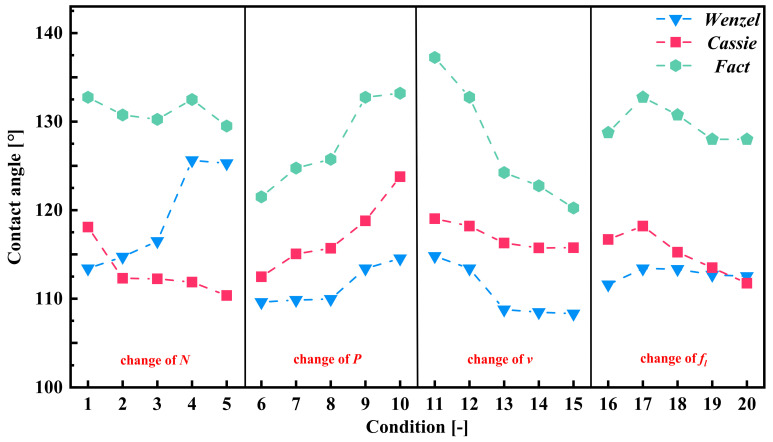
Actual measured static contact angle and the static contact angle predicted by the two models.

The addition of parallel grooves made the textured surface become a wetting anisotropic surface. The contact angle is different when the viewing angle is parallel to the groove direction and the viewing angle is perpendicular to the groove direction. The contact angles of water droplets at different angles of view are shown in Figure 10. A single droplet is located on a smooth surface and a groove-textured surface. The droplet volume is 10 μL, the number of grooves is 25, and the groove spacing is 200 μm. The static contact angle and spreading time of the oil droplets on the surface of each group of samples were measured and recorded along the groove. The CA value of each group of samples is obtained by averaging the two contact angle measurements. Figure 11 shows the schematic diagrams of two wetting models and the calculated roughness factors. Figure 12 shows the actual measured static contact angle and the static contact angle predicted by the two models.

In this study, the predicted θ values will be used for comparison with the results of the widely utilized Wenzel and Cassie–Baxter wetting models [26,27] to explore the wetting mechanism. Equations (2) and (3) represent the Wenzel and Cassie–Baxter relations of micro-textured surfaces, separately [27]. Here, *r*, *f*, $\theta_{Y}$, $\theta_{A}$, and $\theta_{B}$ represent the Wenzel roughness factor, Cassie–Baxter roughness factor, Young’s equilibrium contact angle of texture surface, Wenzel contact angle, and Cassie–Baxter contact angle, respectively [28,29]. In addition, the Wenzel roughness coefficient (*r*) and the Cassie–Baxter roughness coefficient (*f*) are defined as the ratio of the actual wetting surface area to the projected area per unit pitch, and are derived from the geometric representation shown in Figure 11. It can be seen from Figure 11a that, for the Wenzel state, the droplets completely diffuse into the texture geometry and wet the entire region, while the Cassie–Baxter state does not allow the droplets to diffuse to the texture part, because there is retained air in the texture region, resulting in the droplets staying at the top of the texture surface (Figure 10b). The actual wetting surface area and projection area are measured.

(2) $$\cos\theta_{A}=r\cos\theta_{Y}$$

(3) $$\cos\theta_{B}=f(\cos\theta_{Y}+1)-1$$

**Figure 13:**
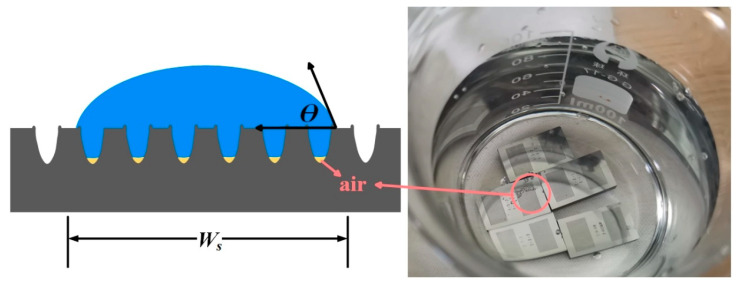
Outflow of residual air in microstructure.

From Figure 10, it is observed that the Wenzel roughness factor is almost consistent with the trend of texture width change, but the roughness factor value of condition 1–5 is similar to that of condition 1–4, which is due to the width and depth of texture not having changed much. From Figure 10a, the initial polished textureless WC/Co cemented carbide surface with Young’s equilibrium contact angle (θ*_Y_*) = 107.9° is essentially symmetrical and exhibits oleophobic behavior. In addition, Figure 12 shows the comparison between the predicted values of the Wenzel contact angle and Cassie–Baxter contact angle parallel to the groove and the actual CA value. It can be seen that the processed surfaces with the groove-shaped texture belong to the oleophobic surface, and the contact angle of the surface is larger than that of the smooth surface, indicating that the oleophobicity increases. These results support the assumption that the surface roughness amplifies the initial wettability of the reference surface. The oil droplets are in the Wenzel state, and the wetting state can be described by the Wenzel model. It can also be clearly observed that, for the groove-textured surface, the equilibrium contact angle increases with the increase in Wenzel surface roughness. In addition, it is observed that the predicted equilibrium contact angle is smaller than the actual equilibrium contact angle, which may be due to the fact that the oil droplets on the groove-textured surface do not completely belong to the Wenzel model. As shown in Figure 13, the sample with oil droplets on the surface is completely immersed in water, and a small number of bubbles is observed at the contact position between the oil droplets and the surface, confirming the above statement. The progress of surface roughness of Cassie–Baxter is the opposite of the trend of texture width, and the predicted contact angle is also smaller than the actual equilibrium contact angle. It also proves that the oil droplets do not completely belong in the Cassie state, and it can be proven that the wetting mechanism of the surface of the textured sample is not only caused by the geometric change of the texture, but is also affected by other factors. Since the samples in this paper are not ultrasonically cleaned after the laser processing of the micro-texture, the continuous burrs formed by the spatter structure of molten particles on the surface of the samples, such as in U. Sudeep’s article [30], have demonstrated that the continuous burrs on the texture surface contribute to its airtight nature, aiding in the enhancement in the SCA. Nevertheless, the formation of burrs will affect the spread of oil droplets on the surface.

**Figure 14:**
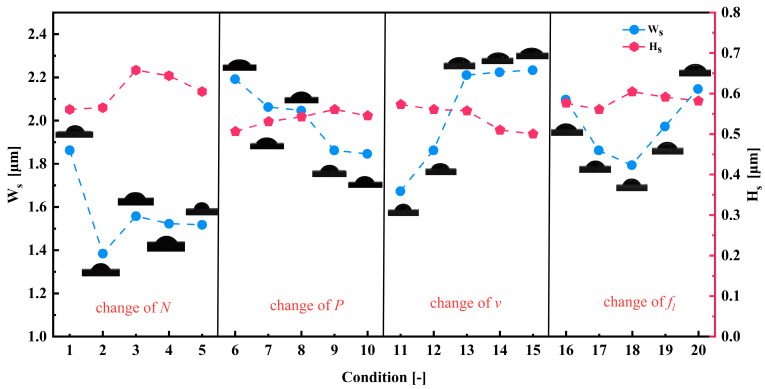
The width and height of oil droplets after spreading 2.5 s on the groove surface.

It can be seen from Figure 14 that, after spreading for 2.5 s, the trend of the droplet width change is opposite that of the groove width. With the increase in groove width, the spreading width of the oil droplets decreases. This is because, when the droplet is in the perpendicular direction of the wetting groove, the three-phase contact line cannot be continuously wetted due to the structural limitation of the groove during the outward wetting process. When the three-phase contact line is crossing the continuous groove, the surface width of the adjacent groove is not large, and the resistance will be encountered when crossing the groove. The contact area between the droplet and the surface will change rapidly, and the resistance received is called the energy barrier. When the groove width increases, the energy barrier of the oil droplets increases, so that the spreading of the oil droplets in the perpendicular direction to the groove is affected.

The reason why the oil droplet width of the second group of samples decreases significantly is that the depth of the texture increases significantly, which leads to the increase in the overall volume of the texture, so that the oil droplets are more immersed in the texture, and the support force of the internal air to the oil droplets is reduced, which makes it more difficult for the oil droplets to spread outward. The enlargement of the oil droplets in conditions 3–4 can be attributed to the narrower and longer texture generated, resulting in a smaller texture volume. This causes the internal air support for the oil droplets to increase. However, the heightened surface burr makes it more challenging for the oil droplets to spread. The oil droplets are more inclined to spread along the groove direction, so the width of the oil droplets perpendicular to the groove direction is also decreasing.

The trend of the height change of the oil droplet spreading on the surface was consistent with change in the groove depth. The spreading ability of the groove texture achieved in a single laser-processing pass was superior when compared to multiple passes. While a small texture width and depth facilitated a good spreading of oil droplets on the surface, the oil transport capacity on the surface was relatively low. Furthermore, the geometric accuracy of the processed texture differed significantly from the intended design size, making precise control challenging during processing.

### 3.3. Influence of Laser-Processing Parameters on Wettability

For example, as Fasasi et al. [31] pointed out, the surface chemical composition of the metal substrate can be changed by laser texturing, mainly through material vaporization and chemical reactions with the atmosphere, mainly oxidation. In order to explore the change in chemical composition on the surface of a WC/Co cemented carbide sample and further link the surface chemistry with wettability, the sample was analyzed by X-ray photoelectron spectroscopy (XPS).

The increase in contact angle can be attributed to changes in surface energy. Materials with a low surface energy have a tendency to accumulate on structured surfaces. As shown in Figure 14 of the XPS results, substances with a potential low surface energy were identified as carbon materials. The carbon content within the groove shows an increase. Post laser treatment, the formation of metal oxides occurs. Metals or metal oxides have the ability to spontaneously attract organic carbon materials from the atmosphere, thus lowering the surface energy [32]. The accumulation of carbon serves as an effective shield for the surface, leading to a substantial increase in contact angle. Consequently, the modification in surface wettability is undoubtedly influenced by both the alteration in surface morphology and composition.

**Figure 15:**
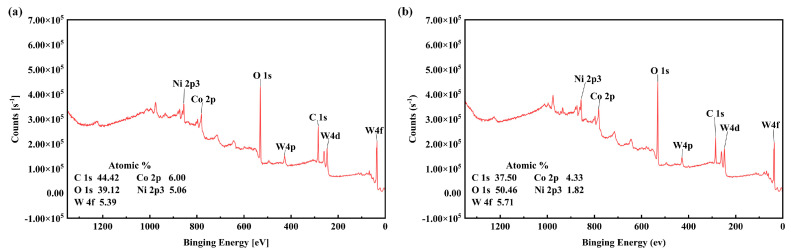
The XPS full spectrum of WC/Co cemented carbide samples textured by laser for two different number of passes: (**a**) 1 pass; and (**b**) 15 passes.

The XPS full spectrum and the corresponding atomic percentage of different elements for the samples used for the surface chemical analysis (castings, dented textures, and recessed textures) were considered, as shown in Figure 15. It can be seen from the XPS full spectrum of the grooved texture samples (Figure 15a) that carbon (38.24%) and oxygen (42.96%) were detected as the main elements, which was attributed to surface contamination and oxidation. In addition, some small peaks in the Cu and Ni regions were also observed on the surface of the groove-textured samples.

**Figure 16:**
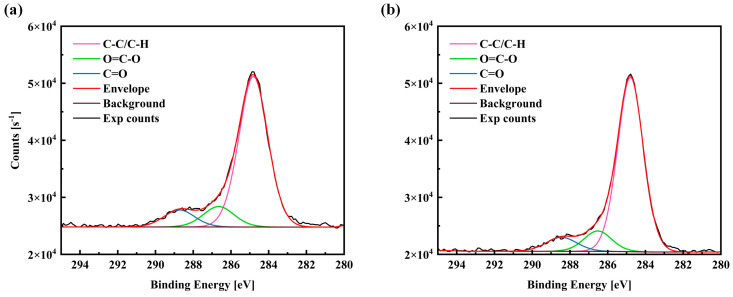
The high-resolution spectra of the C1s region on the surface of WC/Co cemented carbide after 1 pass and 15 passes of laser surface texture treatments: (**a**) 1 pass; (**b**) 15 passes.

The corresponding XPS spectral illustration in Figure 16 shows the detailed atomic element composition of the sample without the texture pattern. These data indicate that the percentage of W and Co concentration on the surface of the sample after laser texture treatment is lower than that of the cast surface, which may be due to material vaporization during laser irradiation. In addition, although the carbon content of the sample was 38.24% after one round of laser scanning, the carbon content increased to 41.96% and 44.21% after 15 and 20 rounds of laser processing, respectively. This means that more hydrocarbons were introduced into the sample surface after the number of laser surface texture treatments increases. In contrast, the oxygen content of the samples decreased from 42.96% to 39.33% and 39.12%, respectively, indicating that the metal oxides were removed from the surface. In addition, the concentrations of Cu and Ni on the surface of all samples were quite low due to oxidation and contamination. In the previous review of the relevant literature, it was reported that the carbon groups on the surface should have a significant effect on wettability. Therefore, a higher-resolution carbon XPS spectrum was also performed. Figure 16 shows these regions of the C1s region. According to this diagram, carbon–carbon (C-C) bonds were observed at 284.8 eV, 286.7 eV, and 288.7 eV on the groove-textured samples, respectively.

In summary, when the number of passes is less than 5, the laser power is about 80% (16 W), the scanning speed is between 100–150 mm/s, and the repetition frequency is 30 kHz, the width and depth of the groove texture on the surface of the cemented carbide were processed. The size is relatively good, and the surface morphology of the workpiece is also relatively good, resulting in a relatively good oleophobic surface and good oil droplet spreading performance.

## 4. Conclusions

In this study, the groove texture was produced on the surface of WC/Co cemented carbide by Nd: YAG laser. The purpose was to study how different laser parameters affect the morphology and wettability of the groove texture, so as to improve the surface texture accuracy and wettability of laser processing. The surface morphology and accuracy of laser-marked samples with different parameters in generating the groove texture were studied and compared. The static contact angle and the droplet size after spreading for 2.5 s were measured, and the wettability of the samples under different groove morphologies was evaluated. Through an XPS analysis, the surface chemical composition after laser texture with different parameters was also compared, trying to explain how the change in surface elements after laser marking contributes to the change in wettability.

(1) The study investigated the surface groove texture processing characteristics of WC/Co cemented carbide using laser processing. The optimal laser parameters identified were: less than 5 passes, a laser power of approximately 16 W, a scanning speed between 100–150 mm/s, and a repetition frequency of 30 kHz. It was found that the surface integrity and precision of the groove texture were the best when these parameters were utilized. The width of the groove texture closely aligned with the laser spot diameter, showing a deviation of only 4.7%

(2) Consistent with previous reports on the application of weaves to surfaces, it was found that grooved weaves can enhance the oleophobicity of surfaces. The output of the laser-generated slag increases surface roughness and surface energy, ultimately improving surface oleophobicity. Additionally, surfaces with morphologically complete and highly precise groove weaves exhibited relatively good surface oleophobicity and oil droplet spreading properties.

(3) Based on the results of the surface morphology and chemical analysis, this increase in oleophobicity can be attributed to the dominant role caused by a higher surface roughness, and the change in surface chemical elements plays an auxiliary role. This result was attributed to the fact that surface chemical modification plays a more important role in this case, especially due to the reduction in hydrophilic carbon-based bonds (C=O) and carboxyl species (O=C-O).

## Figures and Tables

**Table 1:** Mechanical properties of cemented carbide (WC/Co cemented carbide).

Composition	Density	Young’s Modulus	Poisson Ratio	Thermal Expansion Coefficient	Thermal Conductivity	Specific Heat
(wt %)	(kg/m^3^)	(GPa)		10^−6^/K	(W/(m·°C))	(J/(kg⋅K))
WC + 8% Co	14,700	540	0.3	4.5	75.4	470

**Table 2:** Laser parameter factor level table.

*N*	*P*	*v*	*f_p_*
[-]	[W]	[mm/s]	[kHz]
1	4	50	30
5	8	100	40
10	12	150	50
15	16	200	60
20	20	250	70

## Data Availability

The original contributions presented in the study are included in the article, further inquiries can be directed to the corresponding author.
